# Supplementary material for: Causes and consequences of sex-chromosome turnovers in Diptera
Source: Evol Lett. 2026 Mar 12;10(3):253–68. doi: 10.1093/evlett/qrag003 (PMC13231274; doi:10.1093/evlett/qrag003)
Supplement: qrag003_Supplemental_Files [file qrag003_supplemental_files.zip › Supplementary_Datasets.zip!/Supplementary_Datasets/Dataset_2_Graphical_representation_outgroups/Drosophilidae_Drosophila_miranda_chromosomes.pdf]

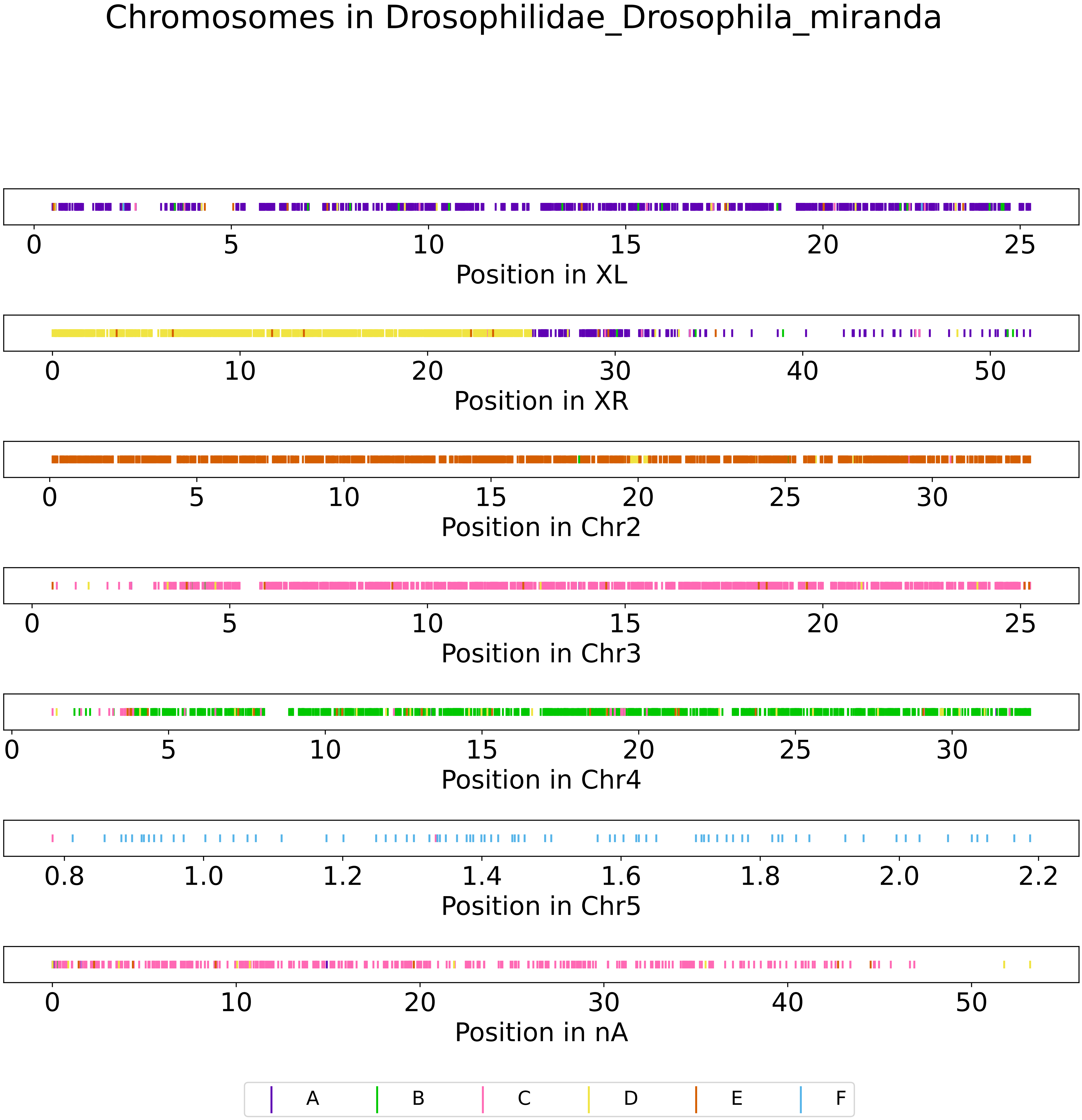

Chromosomes in Drosophilidae_Drosophila_miranda
0
5
10
15
20
25
Position in XL
0
10
20
30
40
50
Position in XR
0
5
10
15
20
25
30
Position in Chr2
0
5
10
15
20
25
Position in Chr3
0
5
10
15
20
25
30
Position in Chr4
0.8
1.0
1.2
1.4
1.6
1.8
2.0
2.2
Position in Chr5
0
10
20
30
40
50
Position in nA
A
B
C
D
E
F
